# Interaction of the GTPase Elongation Factor Like-1 with the Shwachman-Diamond Syndrome Protein and Its Missense Mutations

**DOI:** 10.3390/ijms19124012

**Published:** 2018-12-12

**Authors:** Abril Gijsbers, Diana Carolina Montagut, Alfonso Méndez-Godoy, Davide Altamura, Michele Saviano, Dritan Siliqi, Nuria Sánchez-Puig

**Affiliations:** 1 Departamento de Química de Biomacromoléculas, Instituto de Química, Universidad Nacional Autónoma de México, Circuito Exterior s/n, Ciudad Universitaria, Ciudad de México 04510, Mexico; a.gijsbers@maastrichtuniversity.nl (A.G.); diana.montagut29@gmail.com (D.C.M.); alfmengod.qfb@gmail.com (A.M.-G.); 2 Istituto di Cristallografia, Consiglio Nazionale delle Ricerche, Via G. Amendola 122/O, 70126 Bari, Italy; davide.altamura@ic.cnr.it (D.A.); michele.saviano@ic.cnr.it (M.S.)

**Keywords:** Elongation factor-like 1, Shwachman-Bodian-Diamond Syndrome protein, fluorescence anisotropy, SAXS, mutations

## Abstract

The Shwachman-Diamond Syndrome (SDS) is a disorder arising from mutations in the genes encoding for the Shwachman-Bodian-Diamond Syndrome (SBDS) protein and the GTPase known as Elongation Factor Like-1 (EFL1). Together, these proteins remove the anti-association factor eIF6 from the surface of the pre-60S ribosomal subunit to promote the formation of mature ribosomes. SBDS missense mutations can either destabilize the protein fold or affect surface epitopes. The molecular alterations resulting from the latter remain largely unknown, although some evidence suggest that binding to EFL1 may be affected. We further explored the effect of these SBDS mutations on the interaction with EFL1, and showed that all tested mutations disrupted the binding to EFL1. Binding was either severely weakened or almost abolished, depending on the assessed mutation. In higher eukaryotes, SBDS is essential for development, and lack of the protein results in early lethality. The existence of patients whose only source of SBDS consists of that with surface missense mutations highlights the importance of the interaction with EFL1 for their function. Additionally, we studied the interaction mechanism of the proteins in solution and demonstrated that binding consists of two independent and cooperative events, with domains 2–3 of SBDS directing the initial interaction with EFL1, followed by docking of domain 1. In solution, both proteins exhibited large flexibility and consisted of an ensemble of conformations, as demonstrated by Small Angle X-ray Scattering (SAXS) experiments.

## 1. Introduction

The Shwachman-Diamond Syndrome (SDS, OMIM #260400 and #617941) is an autosomal recessive disease with a broad range of clinical presentations, affecting the skeletal, hepatic, cardiac, and immune and central nervous systems [1], with a 30–40% risk of progression to myelodysplastic syndrome [2]. In most cases, SDS is associated with mutations in the Shwachman-Bodian-Diamond syndrome gene (*SBDS*, OMIM gene #607444), arising by gene conversion with the pseudogene *SBDSP*. Most patients harbour the biallelic pathogenic variants c.183_184TA > CT (K62X) and c.258 + 2T > C (C84fsX3), which result in truncated forms of the protein within its first domain [3]. Less common mutations have also been reported throughout the gene including nonsense mutations, missense mutations, small deletions, indel conversions and splice-site mutations [3,4,5,6,7]. The *SBDS* gene is located in chromosome 7q11, and encodes for a protein structurally organized into three highly conserved domains [8,9,10]. The N-terminal region consists of a unique αβ domain, the central part comprises a three helical bundle, and the C-terminal region presents a ferrodoxin-like domain similar to domain V of the elongation factor EF-2. A small number of patients (<10%) present clinical indications of SDS but do not have pathogenic variants in *SBDS*. Similar clinical features have been reported for mutations in the proteins DNAJC21 [11,12], SRP54 [13], and EFL1 [14,15]. In spite of all being implicated in the cytoplasmic maturation of the 60S ribosomal subunit, only EFL1 participates with SBDS in the same step. EFL1 is a GTPase encoded in chromosome 15q25, comprising 1120 residues (OMIM gene #617538). The protein is organized in 5 domains similar to those observed for EF-2, with domain 1 responsible for guanine nucleotide binding and hydrolysis. Together, EFL1 and SBDS promote the release of eukaryotic initiation factor 6 (eIF6, Tif6 in *Saccharomyces cerevisiae*) [8,16] and assess the integrity of key functional 60S subunit sites, namely, the polypeptide exit tunnel, the P site, the GTPase center, the base of the P-stalk, and the sarcin–ricin loop (SRL). Premature association of the ribosomal subunits is prevented by eIF6 binding to the 60S inter-subunit face [17], and it must be removed to allow the assembly of translational competent ribosomes [16]. SBDS acts as the nucleotide exchange factor (GEF) for EFL1, increasing its affinity for GTP over GDP [18,19]. On the surface of the ribosome, EFL1 binds to GTP and SBDS and displaces eIF6 through competition for an overlapping binding site, followed by GTP hydrolysis and the release of both factors. Mutations in either SBDS or EFL1 prevent the release of eIF6, which results in fewer mature 60S subunits and consequently fewer ribosomes available to engage in translation [14,16]. Cells with mutations in SBDS have impaired general translation rather than specific translation regulation, and not only do they accumulate immature 60S subunits, but their overall pool of 60S subunits is less competent for translation even after they have released eIF6 [20]. Evidence supports the idea that cells with reduced SBDS activity are able to grow and perform as wild type cells in proper conditions, but if SBDS function drops below a certain threshold then the cells display a phenotype corresponding to that of the clinical manifestations of SDS [20]. The same probably also applies for the mutations described in EFL1, but these have not been studied so far.

SBDS missense mutations can be divided into those affecting the stability of the protein, and those that modify surface epitopes without altering the protein fold [8]. Unfolding of SBDS results in limited amounts of protein available to fulfil its function, but the impact that surface modifications may have on the function of SBDS is still largely unknown. We used a combination of biophysical techniques such as fluorescence anisotropy and Small Angle X-ray Scattering (SAXS) to describe the interaction between EFL1 and SBDS, and to study the impact SBDS surface missense mutations have on this interaction. We showed that all the surface missense mutations tested, regardless of their location on the SBDS protein, disrupted the binding to EFL1. In turn, lack of interaction between SBDS and EFL1 may consequently prevent the enzymatic activation of EFL1 required to evict eIF6 from the 60S subunit. Dissection of the binding mechanism showed that the interaction occurs via two cooperative binding events, initially mediated by domains 2–3 of SBDS, followed by an induced conformational change in domain 1. Modelling of the EFL1•SBDS complex by *ab initio* docking aided by the SAXS data suggested that domain 2 of the GTPase is involved in the interaction.

## 2. Results

### 2.1. Interaction between Human EFL1 and SBDS Missense Variants

**Figure 1:**
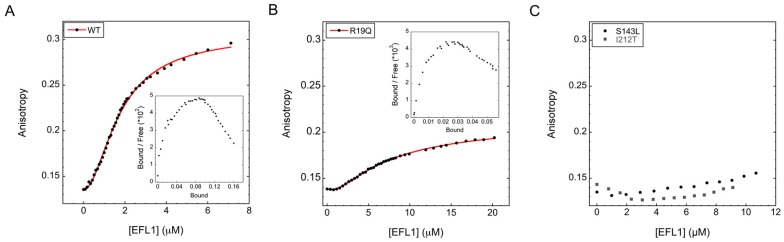
Binding of human Elongation Factor Like-1 (EFL1) to representative Shwachman-Bodian-Diamond Syndrome protein (SBDS) missense disease variants, evaluated by fluorescence anisotropy. (**A**) SBDS wild type, (**B**) SBDS R19Q, (**C**) SBDS S143L and I212T. Continuous line represents the fit of the data to a two different binding sites model. Insets in (A) and (B) correspond to the Scatchard plots.

SBDS missense variants can be classified into two groups: those affecting protein folding and those that modify surface residues [8]. Here, we investigated the effect that changes on the surface of SBDS have on the binding to EFL1 by fluorescence anisotropy, and provided a detailed quantitative and mechanistic description of the binding events. The SBDS variants were labelled with the Lumio™Green dye as fluorescent probe, achieving at least 80% labelling after 8 h reaction. The free dye was removed by dialysis to avoid interferences during the measurements. Association of the wild type proteins displayed a sigmoidal binding curve (Figure 1A), suggesting that more than one binding event was occurring. Data could not be fitted properly to a single binding site model [21], but fitted equally well to a two identical binding sites model and a two distinct binding sites model. A two identical binding sites model, however, was difficult to explain for the interaction between monomeric proteins such as EFL1 and SBDS. SAXS experiments using the yeast orthologues showed that both proteins are monomers in solution (Supplementary Table S1). Analysis of the yeast SBDS (herein referred as Sdo1) demonstrated a radius of gyration (*R_g_*) of 27 Å with a maximal dimension (*D_max_*) of 86 Å, corresponding to a molecular weight of 22 kDa; a value in good agreement with the 29 kDa expected from its sequence. Whereas, for the yeast EFL1, the calculated value corresponded to an *R_g_* = 46 Å with a *D_max_* = 157 Å and a molecular weight of 136 kDa, which compares well with the slightly smaller value of 129 kDa calculated from its amino acid sequence. On the other hand, a binding model corresponding to two different binding sites can be easily reconciled for monomeric multi-domain proteins such as EFL1 and SBDS. Fitting of the data to this model resulted in dissociation constants of 5 µM and 0.4 µM for the first and second binding events, respectively. The higher affinity of the second binding site suggested a positive cooperative effect, which was confirmed by the 1.8 value for the Hill coefficient obtained from the Hill plot analysis and the concave-down curve in the Scatchard plot (Figure 1A inset). This same analysis was performed for the SDS disease surface missense variants present in the different domains of SBDS. In all cases, a two distinct binding sites model with positive cooperativity best described the interaction. Results are summarized in Table 1, and representative examples of the binding curves corresponding to mutations in each domain of SBDS are shown in Figure 1B,C. All the mutations tested debilitated the interaction with EFL1. However not all mutants behaved equally; some mutations completely abolished the interaction with EFL1 while others only weakened it, but all had a detrimental effect on the binding. Mutants R19Q and K67E showed an order of magnitude decrease in the affinity of the second binding event, while the dissociation constant for the first binding remained unchanged within the experimental error. These two residues locate to domain 1 of SBDS, suggesting that the second binding site maps to this region of the protein. On the other hand, binding of EFL1 to the missense variants R126T and I167T contained within domain 2 of SBDS resulted in both binding sites affected. Most likely, loss of binding in the first site negatively impacts the binding on the second site by cooperative effect. Thus, the first binding event most likely occurs in domain 2 of SBDS. The other domain 2 mutant, S143L, weakened the binding to such an extent that it was not possible to reach ligand saturation, and data could not be fitted to obtain the corresponding dissociation constants. Surprisingly the missense mutations R175W and I212T contained in domain 3 of SBDS [22] also severely disrupted the binding to EFL1, suggesting that this domain also plays a role in the interaction. The behaviour of the variant R175W resembled that of variants in domain 2 affecting both binding events. The studied mutants exhibited differences in their Hill coefficients, however it is difficult to relate these variations to changes in the cooperativity mechanism, since the Hill coefficient is a macroscopic measurement that do not provide molecular information. Decrease in affinity clearly resulted from the missense mutations in SBDS, and not because of lack of proper protein folding. The secondary and tertiary structure content of all the studied mutants have been previously corroborated by Finch and collaborators using NMR techniques [8].

### 2.2. Interaction between Human EFL1 and the Different Domains of SBDS

**Figure 2:**
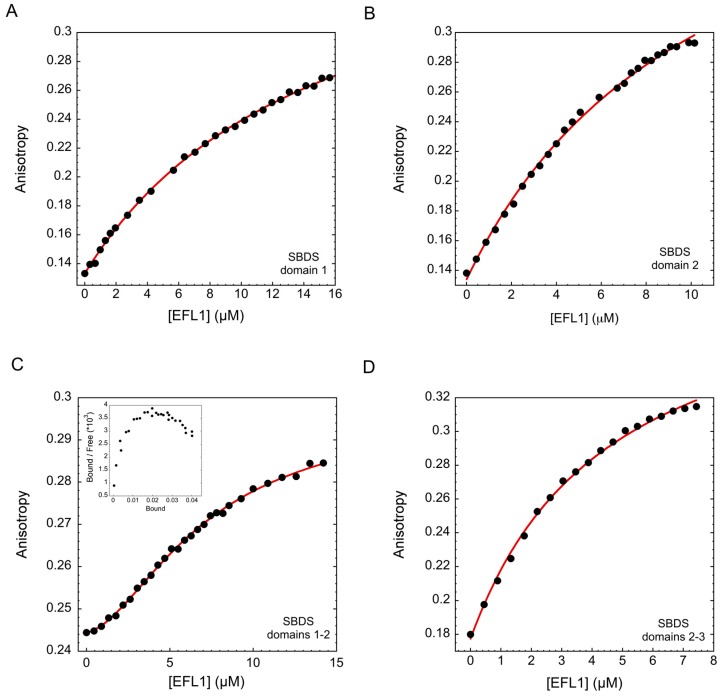
Binding of human EFL1 to different domains of human SBDS, evaluated by fluorescence anisotropy. (**A**) Domain 1, (**B**) Domain 2, (**C**) Domains 1–2, (**D**) Domains 2–3. Continuous line represents the fit of the data to a single binding site model except for plot (C), where data were fitted to a two different binding sites model. Inset in (C) corresponds to the Scatchard plot.

Results from the previous section established that two different binding sites exist in SBDS that contact EFL1, however, structurally SBDS consists of three well-defined domains and all of them seem to participate in the interaction. To understand this observation, we dissected the SBDS protein to its different domains and combinations thereof, and studied their binding to EFL1 (Figure 2 and Table 1). Interaction of EFL1 with domain 1 of SBDS (residues 1–94) resulted in a hyperbolic curve that was best described by a single binding site model. The dissociation constant corresponded to 15 µM, a value forty times larger than that observed for the second binding event of the full-length protein. This difference may have arisen from the loss in cooperativity due to the absence of the first binding site of the protein. The interaction with domain 2 also corresponded to a one binding site model, with a dissociation constant of 11 µM. This value is slightly larger than the dissociation constant of 5 µM obtained for the first binding event for the wild-type protein. This indicates that the first binding event did not exclusively occur in domain 2 and the third domain must also have participated in the binding, as suggested by the domain 3 mutants I212T and R175W. Interestingly, the dissociation constant for a construct consisting of domains 2–3 of SBDS corresponded to 4.6 µM, a more avid interaction than that of domain 2 alone, which can only be conferred by the presence of domain 3. This interaction however, was described by a one binding site model, implying that domains 2–3 of SBDS most likely bind to EFL1 as a single entity through a rigid body interaction. Finally, testing the interaction of a construct consisting of domains 1–2 of SBDS to EFL1 resulted in a sigmoidal curve similar to that previously observed for the full-length protein. Fitting the data to a two different binding site model resulted in values for *K*_*d*1_ and *K*_*d*2_ of 11 µM and 4 µM, which were larger than those for the wild-type protein because of the missing contribution of domain 3. Noteworthy is the almost identical value of the dissociation constant for the first binding event of this construct, attributed to domain 2, with that of the isolated domain 2. Furthermore, the *K_d_* for the isolated construct of domain 2–3 was also very similar to the first binding site of the wild-type protein. Despite the absence of domain 3, this construct still elicited positive cooperativity for binding to EFL1 with a Hill coefficient of 1.4 (Table 1) and a concave downward Scatchard plot (inset Figure 2C). In light of these results, we could envisage a model with SBDS initially contacting EFL1 through domains 2–3, followed by docking of domain 1 facilitated by the flexible linker that joins it with the rest of the protein.

### 2.3. Small Angle X-ray Scattering and Flexibility Analysis of the Yeast SBDS, EFL1 and the EFL1•SBDS Complex

**Figure 3:**
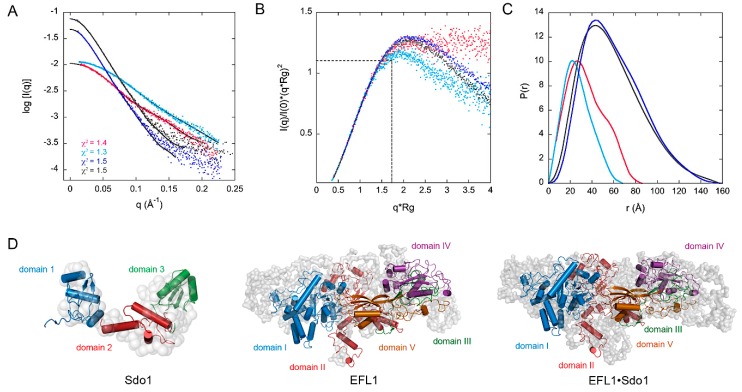
Solution structure of the yeast proteins Sdo1 and EFL1 alone and in complex. (**A**) Experimental scattering curves compared to the fit (solid traces) of the simulated curves calculated from homology models. (**B**) Dimensionless Kratky plot. The intersection of the dotted black trace corresponds to the value for bovine serum albumin. (**C**) Pair-distance distribution function plot, P(r). Color codes correspond to magenta—Sdo1, light blue—Sdo1 domains 2–3, grey—EFL1, and dark blue—Sdo1•EFL1 complex. (**D**) Low resolution Small Angle X-ray Scattering (SAXS) envelopes of the studied proteins, showing the corresponding fitted homology models.

We used Small Angle X-ray Scattering (SAXS) to probe the structure of the yeast proteins Sdo1, EFL1, and the complex in solution, and to study their flexibility (Figure 3 and Supplementary Table S1). The complex is composed of one molecule of Sdo1 and one molecule of EFL1, with an *R_g_* value of 51 Å and a *D_max_* of 160 Å. The EFL1 protein and the complex appeared as compact but asymmetric particles, as evidenced by the shift in the observed maxima towards values larger than 1.7 in the dimensionless Kratky plot (Figure 3B). The maximum value of 1.1 at $\sqrt{3}$ for q*R_g_ corresponds to a globular and compact protein, similar to the bovine serum albumin (BSA) protein used as a standard in the experiment. The Sdo1 curve displayed a hyperbolic plateau suggesting a highly flexible protein, as has been previously described for the *Archaeoglobus fulgidus* SBDS [23]. Interestingly, deletion of domain 1 in Sdo1 resulted in a Kratky plot characteristic of a compact protein. Inspection of the scattering element distribution described by the *P(r)* function curve for EFL1 and the complex confirmed that the molecules had elongated shapes and very similar maximal dimensions (Supplementary Table S1). In contrast, that for the full-length Sdo1 showed a pronounced shoulder at 50 Å that was not present in the construct lacking domain 1. This indicates not only an elongated protein, but also the presence of distinct domains joined by flexible linkers and possible different conformations (Figure 3C). Thus, Sdo1 is an elongated molecule with a flexible region consisting of domain 1, which agrees with the fluorescence anisotropy results presented in the previous section. Low-resolution *ab initio* models of the three-dimensional arrangement described by the experimental SAXS data provided information of the molecular envelope of the studied proteins (Figure 3D). Comparison of the measured data with that of the calculated scattering profiles of homology models showed a good agreement, as evidenced by the chi square values for Sdo1, EFL1, and the complex, respectively (Figure 3A black trace). The homology model for EFL1 was obtained using the crystallographic structure of yeast EF-2 (PDB:1N0U [24]), which in turn served as a template to model human EFL1 bound to 60S ribosomal subunits in Cryo-Electron Microscopy (Cryo-EM ) studies [25]. However, the solution structure of EFL1, alone or in complex with Sdo1, was larger than that observed in the cryo-EM structures, despite human and yeast orthologues both having the same number of residues. The size of the human EFL1 molecule bound to the 60S subunit (PDB:5ANC) had a *D_max_* of 130 Å and *R_g_* = 37 Å (χ^2^ = 31 calculated respect to the experimental data), compared to the *D_max_* of 157 Å and *R_g_* = 46 Å of the protein alone in solution. Not only was EFL1 larger in solution, but so was the complex formed between EFL1•Sdo1 with a *D_max_* = 160 Å and *R_g_* = 51 Å, compared to the respective values of 143 and 41 Å in the presence of the 60S subunit (χ^2^ = 10). Furthermore, to fit the EFL1 model onto the SAXS envelope it was necessary to allow the free rotation of the rigid parts of the protein corresponding to domains 1–2, 3 and 4–5, using residues 673 and 749 as hinges. This reinforced the idea that the free and ribosome-bound forms of EFL1 adopt different conformations. Thus, both proteins, EFL1 and SBDS, undergo large conformational changes upon binding to each other in solution and as a complex, yet they adopt a different conformation when bound to the ribosomal subunit.

**Figure 4:**
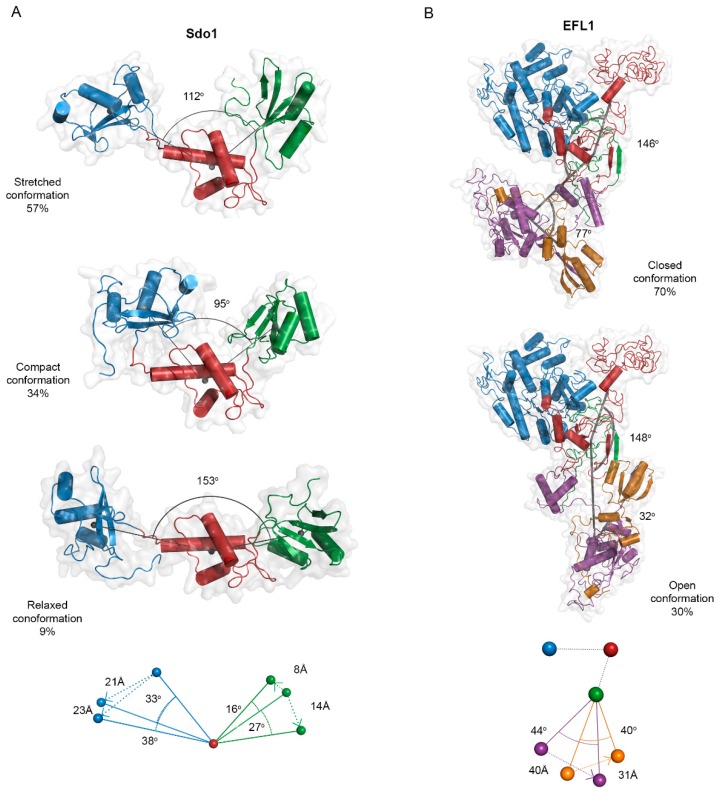
Ensemble of conformations for the yeast proteins Sdo1 and EFL1 in solution. (**A**) Conformational ensemble for Sdo1 in solution and their abundance percentage. Depicted angles show the movement of domain 1 (blue) and 3 (green) respective to the center of mass of domain 2 (red) within the same conformation. Bottom scheme compares the movement of these domains in the three conformations. Using as reference the position of domain 2 in the compact conformation, domain 1 moved downward in both the stretched and relaxed conformation, while domain 3 moved vertically in opposite directions in the stretched and relaxed conformations. (**B**) Conformational ensemble for the yeast EFL1 in solution and their abundance percentage. The two depicted angles show the movements of domains II (red), III (green), and IV (purple), and domains III (green), IV (purple) and V (gold) compared to domain I (blue) within the same conformation. Using as reference the position of domains I, II, and III, the bottom scheme compares the movement of domain IV and V.

In light of these results, we further studied the flexibility of Sdo1 and EFL1 using the SAXS information. We used the program MultiFOXS (web version http://modbase.compbio.ucsf.edu/multifoxs/ [26]) to analyse the flexibility of Sdo1 and EFL1 (Figure 4). The program uses the atomic structure of the studied protein, a list of flexible residues (described in the methodology section for EFL1 and Sdo1), and the corresponding experimental SAXS profile. It generates 10,000 conformations and their corresponding calculated SAXS profile, followed by enumeration of the best ranked state models according to a multi-state scoring function (χ). This enumeration of multi-state models corresponds to the sum of weighted calculated SAXS profiles fitted to the experimental SAXS data [26]. The weighted values describe the abundance of each conformation in a multi-state model. Supplementary Table S2 and Supplementary Figure S1 show the *R_g_* distribution for the top 100 best scoring state models for both proteins, in which *state n* (*n* = 1–4) means the state described by *n* conformations. Analysis for Sdo1 resulted in an ensemble constituted by a 4-state model. The three- and four-state models comprise conformations with similar *R_g_* and similar relative abundance that differ from those described in the 2-state model by the presence of an extended conformation (Supplementary Table S2). We selected the three-state models, since a similar flexibility analysis for the *A. fulgidus* SBDS using the program EOM also predicted such a conformational ensemble [23], and contained the most populated conformations at 27–29 Å. This ensemble comprised a ‘compact’ conformation with an *R_g_* of 24 Å, a ’stretched’ one with *R_g_* of 28 Å, and a ‘relaxed’ conformation having an *R_g_* of 30.4 Å. Increase in the *R_g_* compared to that of the compact conformation results from an increase in the angle formed between domains 1 and 3, respective to the centre of mass of domain 2. In the stretched conformation, domain 1 was displaced 33° and 21 Å downwards, while domain 3 shifted in the upward direction 16° and 8 Å, compensating the distance between the two ends of the protein. For the most open relaxed conformation, domain 1 underwent a larger rotation (38° and 23 Å), accompanied by movement of domain 3 in the same direction by 27° and 14 Å (Figure 4A). Additionally, this analysis also established that domain 1 suffers larger movements compared to those of domain 3, suggesting a larger flexibility for the linker between domains 1–2 than that for domains 2–3. The same analysis for EFL1 also converged in an ensemble of 4-state models. The two-, three- and four-state models had similar distributions with two main conformations: one highly populated with *R_g_* between 39–41 Å and a second of 45 Å. Two conformations have also been described for the crystallographic structure of yeast EF-2; a homologue translocase to EFL1 [24]. For these reasons, we selected the 2-state model consisting of one closed conformation with an *R_g_* of 42 Å, and a second open conformation with a larger *R_g_* of 45 Å. The two conformations mainly differed in the orientation of domains IV–V. In the open conformation, domain IV underwent a movement of 40 Å and 44° respective to domain III, while domain V moved 31 Å and 40°, respectively (Figure 4B).

**Figure 5:**
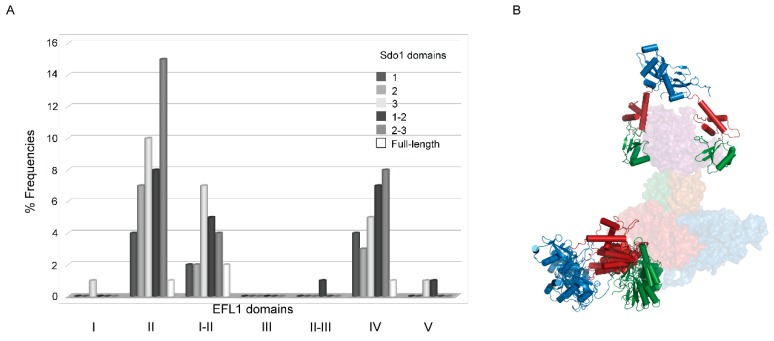
Modelling of the yeast EFL1•Sdo1 complex based on the SAXS data. (**A**) Frequency of contacts between the different domains of the Sdo1 and EFL1 yeast proteins for the best 100 docking models. (**B**) Structure of the best 10 models, showing the interaction between the Sdo1 and EFL1 proteins and their relative positions in the complex. Sdo1 (ribbons) domain color code: blue—domain 1, red—domain 2, green—domain 3. EFL1 (surface) domain color code: blue—domain I, red—domain II, green—domain III, purple—domain IV, gold—domain V.

Due to the low resolution of the SAXS data (38 Å for the *ab initio* model, Supplementary Table S1), and the discrepancies in the overall shape of the EFL1•Sdo1 complex in solution with that bound to the 60S subunit, it was difficult to fit the Sdo1 protein onto the SAXS envelope of the complex. To gain insights into the structure of the complex, we used the SAXS experimental data in combination with a protein–protein modelling approach [27]. We used as input files for both proteins the structural coordinates of the 1-state model previously generated from the flexibility analysis, which represented the average of all the conformations present in solution (Supplementary Table S2). The program ranked the best 100 docking models according to their combined figure of merit [27]. Figure 5A shows the distribution of the top one hundred conformations, respective to the domains involved in the interaction. Accordingly, 69% of the models consist of complexes described by the interaction between domains 1, 2, and 3, and combinations thereof, of Sdo1 with domain I or II of EFL1. Interestingly, in this pool of models most of the positions adopted similar conformations, with only domain 1 not making contact with EFL1, because the program could not take into account the flexibility of this domain (Figure 5B). The rest of the conformations are described by interactions occurring in domain IV, and to a lesser extent in domain V, of EFL1 with any of the three domains of Sdo1. Contained in this 31% of modelled conformations, the interaction postures varied greatly without an apparent consensus, and the contacts differed even within regions of the same domain. In all, the results from the docking of the EFL1•Sdo1 complex to the experimental SAXS information suggests that the interaction sites for the complex occur in domain II of EFL1, and any of the three domains of Sdo1. This information not only parallels that presented above from the fluorescence anisotropy measurements, but also further information obtained by yeast two-hybrid experiments (Supplementary Figure S2). Evidence using as bait two constructs of Sdo1, one consisting of domains 2–3 and another consisting of the full-length protein, suggested an interaction between domains I–II and domain II, but not domain I alone, of EFL1 with the three domains of Sdo1. The measured signal was higher when using the complete Sdo1 compared to that of the truncated version, consistent with the idea that domain 1 of Sdo1 constitutes a secondary binding site that strengthens the interaction.

## 3. Discussion

### 3.1. Molecular Function of the SBDS Missense Mutations on SDS

The Shwachman-Diamond syndrome (SDS) is an autosomal recessive disorder that generally presents in early childhood. Molecular diagnosis is normally established by documenting biallelic homozygosity or compound heterozygosity mutations in the *SBDS* gene. The most common mutations include 258 + 2 T > C and 183–184 TA > CT, which produce the truncated versions of the protein K62X and C84fsX3, respectively [3]. At the molecular level, the majority of SBDS mutations result in decreased or undetectable amounts of the protein, depending on the tissue tested and the mutation involved [28,29]. Homozygosity for the mutation 258 + 2T > C SBDS (C84fsX3) presents low levels of SBDS expression, due to correct residual splicing of the transcript that otherwise would be lethal due to the lack of protein. This also applies for compound heterozygotes consisting of the aforementioned mutation together with the other frameshift mutation 258 + 2 T > C (K62X) or missense mutations. Missense mutations fall into two categories: those that destabilize the SBDS protein fold resulting in the absence of protein, as it would be for frameshift mutations that truncate the protein, and those that modify surface epitopes without altering the stability of the protein. Because SBDS has been reported to be the guanine nucleotide exchange factor (GEF) for EFL1 [18,19], it is expected that decreased amounts of SBDS may not be sufficient to activate EFL1 at the levels necessary to elicit the required physiological effect. The report of a compound heterozygous patient with the mutations SBDS N121T/R175W, consisting of a stability and a surface mutation [30] respectively, suggests that not only is insufficient protein detrimental for the activity of SBDS, but also for recognition of ligands through its surface epitopes. With this in mind, we further investigated the molecular interaction between EFL1 and SBDS wild type and surface missense mutations. All the missense variants tested in this work perturbed the interaction between SBDS and EFL1 to different extents. Some mutations, like S143L and I212T, almost completely abolished the binding between the proteins. Decreased binding, in turn, will result in not enough EFL1 bound to SBDS to promote the exchange of GDP for GTP necessary to activate catalysis. Previous evidence in the literature showed that the SBDS missense mutations R126T and K151N are not able to stimulate the GTPase activity of EFL1 in the presence of 60S subunits [8]. Although these surface missense mutations do not affect protein levels, it seems they lead to the same cellular defect as the most common nonsense SBDS mutations: insufficient SBSD to catalytically activate EFL1 and consequently release eIF6 from the 60S surface. This idea is further supported by the report of compound heterozygous patients with the SBDS mutations N121T/R175W [30] or R175W/K62X (personal communication Akiko Shimamura, Dana Farber/Boston Children’s Cancer and Blood Disorder Center), whose only source of SBDS protein corresponds to that with the mutation R175W. Interestingly none of the reported SBDS surface missense mutations, or those tested here, lie in close proximity to EFL1 in the cryo-EM structures when bound to the 60S subunit [25]. In these models, domain 1 of SBDS points away from EFL1 with no possibility of interaction, instead contacting the 25S rRNA, suggesting these mutations may affect binding to this region rather than to EFL1. In contrast, a report by Holding et al., using the Sdo1 protein substituted with p-benzoyl phenylalanine at specific positions, showed a crosslink between residue S143 of Sdo1 and residue M435 of yeast EFL1 (residue A439 in the human orthologue) [31]. These residues locate to domain 2 of Sdo1 and domain II of EFL1, confirming the participation of these regions in the interaction as evidenced by the results presented here. These discrepancies can only be reconciled by considering that the EFL1•SBDS complex in solution exists in a conformation that undergoes a large rearrangement upon binding to the ribosomal 60S subunit. Some reports have established that a population of SBDS exists in the nucle(ol)us of both human and yeast cells [20,32,33]. Although this may suggest that SBDS potentially binds to the 60S in this compartment and comes out of the nucleus loaded in the subunit, no evidence for this exists. Furthermore, each pool of protein may have different roles, as has been previously described for eIF6. For instance, a reduction of cytoplasmic eIF6 prevents the cells from upregulating protein synthesis, but in the nucleolus a small amount of eIF6 is sufficient for rRNA biogenesis [34]. Additionally, SBDS and EFL1 can bind to mature 60S subunits independently, but SBDS detaches from them at lower ionic strength compared to EFL1 [8,25] and it is well known that electrostatic interactions are crucial for specific binding to nucleic acids [35,36]. Additionally, if EFL1 emulates the behavior of EF-G/EF-2 and other translation GTPases [37], it most likely adopts a GTP-bound active conformation to interact with the ribosomal subunit, and this conformation is attained by the interaction with SBDS. Most likely, they interact with the 60S subunit as a heterodimer. However, in the presence of SBDS missense mutations that abolish the interaction with EFL1, most EFL1 would exist free in the cytoplasm, either in the apo- or GDP-bound form.

### 3.2. Interaction Mechanism between EFL1 and SBDS

A two different binding sites model best described the interaction of EFL1 to SBDS wild type and missense mutations. For the native proteins, fitting of the data demonstrated that after the initial binding event, the subsequent interaction step resulted in enhanced affinity, that is, *K*_*d*2_ < *K*_*d*1_. Further analysis corroborated that the interaction was characterized by positive cooperativity. Because EFL1 and SBDS are monomeric proteins, this cooperative effect can only occur through a conformational change in the complex that accommodates the additional binding event without excessive strain [38]. Clues about the mechanism and conformational changes undergone came from analysis of the dissociation constants of the missense SBDS variants and isolated constructs, and the SAXS experiments. Mutations in domain 1 of SBDS perturbed the second binding but it still occurred through a positive cooperative event, while binding to the isolated domain 1 was weaker than that for the wild type protein. This suggests that domain 1 of SBDS constitutes the region undergoing the conformational change, and that its interaction becomes avid by ‘chelate’ effect with domains 2–3 of the protein. Therefore, SBDS uses all its domains to contact EFL1, with domain 1 and domains 2–3 acting as independent entities. SAXS analyses further support this idea, as they showed that SBDS coexists in several conformations in solution, with domain 1 being the most flexible region while domains 2–3 are fairly rigid. These results were also corroborated by the yeast two-hybrid experiments that showed domains 2–3 of SBDS were sufficient to interact with EFL1, but binding was substantially favoured in the presence of domain 1. Previous reports by Asano and collaborators [39] could not detect binding of the isolated domains of SBDS to EFL1, but suggested domains 2–3 of SBDS to be necessary for binding. From the EFL1 perspective, the N-terminal half of the protein is required for binding to SBDS. This region comprises the GTPase domain and an insertion in domain 2 that characterizes the EFL1 protein family, and would be consistent with the role of SBDS acting as a guanine exchange factor for EFL1 [18,19]. Experiments using isothermal titration calorimetry (ITC) have shown that this insertion in EFL1 was necessary for the interaction with SBDS [39].

The downstream alterations in SDS result from the inability to release eIF6 from the 60S surface due to insufficient amounts of SBDS to favour EFL1 in the GTP bound conformation. This in turn, negatively affects the amount of 60S ribosomal subunits available to be incorporated into active ribosomes, downregulating overall translation. Less SBDS available to perform its function can arise from mutations leading to haploinsufficiency or, as shown here, can be due to mutations that weaken the interaction with EFL1. In spite of this alteration, the SBDS missense mutations have a residual activity, as their interaction with EFL1 is not completely abolished; this may be sufficient to fulfil the physiological requirements of the cells under normal growth, but becomes insufficient under certain conditions.

## 4. Materials and Methods

### 4.1. Protein Expression and Purification

Human SBDS protein wild-type, mutants, truncated constructs, and combinations thereof, as well as the yeast SBDS orthologue (Sdo1), were expressed from a pRSET (ThermoFisher Scientific) plasmid in *Escherichia coli* C41 and purified by conventional chromatographic techniques as described in References [10,21]. Human SBDS and yeast Sdo1 truncated constructs, and combinations thereof, corresponded to domain 1—residues 1–94, domain 2—residues 94–175, domains 1–2—residues 1–174, and domain 2–3—residues 94–250. For the anisotropy experiments, human SBDS and all the variants tested were modified to encode for a C-terminal FlAsH tag corresponding to residues Cys–Cys–Pro–Gly–Cys–Cys. Yeast and human EFL1 were expressed in *S. cerevisiae* BCY123, using the backbone of the pRS426 vector under the control of the *GAL1/10* promoter and the 3’UTR *MATA*, and were purified as described in Reference [21]. The expressed GTPases consist of the corresponding protein fused to a TEV recognition site and a hexahistidine tag at the C-terminus.

### 4.2. Fluorescence Anisotropy Experiments

Fluorescence anisotropy experiments were done with the human SBDS and EFL1 orthologues using an OLIS DM45 spectrofluorimeter (Olis Global Inc., Bogart, GA, USA) equipped with a polarization toolbox. Sample buffer consisted of 50 mM Tris-HCl pH 8, 300 mM NaCl, 5 mM β-mercaptoethanol. Excitation and emission wavelengths were set at 495 and 540 nm, respectively, with a spectral resolution of 8 nm. Anisotropy was recorded after titration of 2 µL additions of 40–90 µM human EFL1 to 300 µL solution of the corresponding 0.1 µM human labelled SBDS-FlAsH construct at 25 °C. To label the SBDS proteins, 3 nmol of each protein was mixed with 6 nmol of 4’, 5’-bis(1,3,2-dithioarsolan-2-yl) fluorescein dye (Lumio™Green) for 8 h at 4 °C. Samples were dialyzed overnight in the buffer mentioned above to remove the free dye. Labelling efficiency was calculated as described in Reference [21], by recording the absorbance of the labelled protein at 280 nm and 508 nm. Data were analysed by nonlinear regression to a single-site or two different binding sites model, depending on the data, with the program KaleidaGraph 4.1 (Synergy Software).

### 4.3. Small Angle X-ray Scattering (SAXS) Experiments

Structural characterization of the yeast proteins was performed by Small Angle X-ray Scattering (SAXS) coupled to an on-line size exclusion chromatography. Data for Sdo1, EFL1 and the complex EFL1•Sdo1 were collected in the bioSAXS beamline B21 at Diamond Light Source, Harwell, United Kingdom. Protein samples consisted of 45 µL of each protein at concentrations of 10, 6.5, and 8 mg mL^−1^, respectively. They were injected onto a Shodex KW-403 size-exclusion column equilibrated with a buffer containing 50 mM Tris pH 8.0, 10% glycerol, 300 mM NaCl, and 5 mM MgCl_2_. The output flow from the Agilent HPLC was directed through a 1.6 mm diameter quartz capillary cell held in vacuum. The flow rate was set to 0.08 mL min^−1^ and 580 frames (with 3 s exposure time) were collected using a PILATUS 2M (Dectris, Baden-Daettwil, Switzerland) detector at a distance of 4.014 m from the sample. Collected two-dimensional images were corrected for variations in beam current, normalized for exposure time, and processed into one-dimensional scattering curves using GDA and the DAWN software (Diamond Light Source, UK). Data for the protein Sdo1 domains 2–3 were collected in the bioSAXS beamline P12-EMBL at DESY Light Source, Hamburg, Germany [40]. Sample consisted of 50 µL at a concentration of 15 mg mL^−1^ and was injected onto a Superdex 200 Increase 10/300 GL size-exclusion column equilibrated with a buffer containing 50 mM Tris pH 8.0, 10% glycerol, 300 mM NaCl and 5 mM MgCl_2_. The output from the FPLC–Malvern TDA system was directed through a quartz capillary cell (50 µm thick walls and a 1.7 mm path length) held in vacuum [41]. The flow rate was set to 0.4 mL min^−1^ and 1850 frames (with 1 s exposure time) were collected using a PILATUS 2M detector at the distance of 3.0 m from the sample. Collected two-dimensional images were corrected for variations in beam current, normalized for exposure time, and processed into one-dimensional scattering curves using integrated software at the beamline [42]. Background was manually subtracted using the program ScÅtter (http://www.bioisis.net/scatter). All the SAXS data were deposited in the Small Angle Scattering Biological Data Bank (www.sasbdb.org) with the following accession codes: SASDEW8 (Sdo1 domains 2-3), SASDEV8 (Sdo1), SASDEU8 (EFL1) and SASDET8 (complex EFL1•Sdo1).

### 4.4. SAXS Data Modelling and Flexibility Analysis

Inspection of the one-dimensional SAXS experimental curves was initially performed to judge the quality of the data. The Guinier plots were used to calculate the zero-angle scattering intensity, *I(0)*, and the radius of gyration (*R_g_*). The latter was calculated from the slope of the Guinier plot in the range of 0–1/*R_g_* [43]. The particle distance distribution P(r) function and maximal dimension of the proteins (*D_max_*) were obtained using the Indirect Fourier Transformation method described in Reference [44]. Additionally, the molecular mass of the proteins was estimated as half (x0.53) of its Porod volume (excluded volume of the hydrated protein particle). Data collection and SAXS parameters for the yeast proteins Sdo1, Sdo1 domains 2–3, EFL1 and the complex EFL1•Sdo1 are listed in the Supplementary Table S1. Low-resolution structures were constructed by *ab initio* modelling using the program GASBOR [45] by aligning, averaging, and filtering ten independently calculated dummy residue models [46]. Experimental data suggest highly ambiguous models for Sdo1 and Sdo1 domains 2–3, and less ambiguous models for EFL1 and the EFL1•Sdo1 complex. Flexibility studies for the yeast proteins Sdo1 and EFL1 were performed using the online version of the program MultiFoXS [26], using as starting models those predicted by the I-TASSER web server [47]. The rigid parts of the proteins were identified using the HingeProt program [48] and corresponded to the three domains of Sdo1 used for the recombinant proteins. In the case of EFL1, the rigid parts of the protein were predicted to be domains 1–2 (residues 1–673), domain 3 (residues 674–747) and domains 4–5 (residues 748–1110).

## Figures and Tables

**Table 1:** Dissociation constants for the interaction between the human EFL1 and different SBDS domains and missense variants.

**SBDS Missense Variants**			
**SBDS**	***K*_*d*1_ (µM)**	***K*_*d*2_ (µM)**	**Hill coefficient**
Wild type 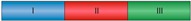	5.4 ± 1	0.4 ± 0.04	1.8 ± 0.02
R19Q 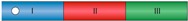	4.2 ± 0.5	5.2 ± 0.3	1.5 ± 0.02
K67E 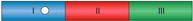	7.2 ± 2	3 ± 1	1.8 ± 0.07
R126T 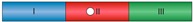	9.3 ± 1.7	2.5 ± 0.2	1.7 ± 0.04
S143L 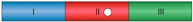	ND	ND	ND
I167T 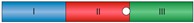	10 ± 3	1.4 ± 0.2	1.5 ± 0.04
R175W 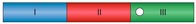	12 ± 4	3.3 ± 0.6	1.3 ± 0.03
I212T 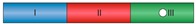	ND	ND	ND
**SBDS Constructs**			
**SBDS**	***K*_*d*1_ (µM)**	***K*_*d*2_ (µM)**	**Hill coefficient**
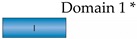	15.2 ± 0.72	-	-
Domain 2 * 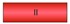	10.8 ± 0.9	-	-
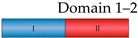	11.3 ± 1.8	3.9 ± 0.2	1.4 ± 0.02
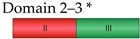	4.6 ± 0.3	-	-

ND—not determined due to a lack of sufficient binding. * Data were fitted to a single binding site model, so there is no second binding event or cooperativity. White dots depicted in the schematic representation of SBDS indicate the relative position of the corresponding missense variant.

## Supplementary Materials

Supplementary materials can be found at http://www.mdpi.com/1422-0067/19/12/4012/s1, Figure S1: Distribution of the radius of gyration (*R_g_*) in the initial pool of random structures of Sdo1 (A) and yeast EFL1 (B) overlapped to those of the final conformational sub-ensembles; Figure S2: Binding of the wild type Sdo1 (A) and a construct of Sdo1 consisting of domains 2-3 (B) to the different domains of yeast EFL1 evaluated by yeast two hybrid; Table S1: SAXS data collection and experimental parameters for the yeast proteins Sdo1, EFL1 and the complex EFL1•Sdo1; Table S2: Ensemble organization of the top 100 best scoring *state n* models represented by the SAXS data for the yeast proteins Sdo1 and EFL1.

## Abbreviations

EFL1	Elongation Factor like-1 protein
SBDS	Shwachman-Bodian-Diamond Syndrome protein
SDS	Shwachman-Diamond Syndrome
eIF6	eukaryotic Initiation Factor 6
SAXS	Small-Angle X-ray Scattering
