# Supplementary material for: Interaction of the GTPase Elongation Factor Like-1 with the Shwachman-Diamond Syndrome Protein and Its Missense Mutations
Source: Int J Mol Sci. 2018 Dec 12;19(12):4012. doi: 10.3390/ijms19124012 (PMC6321010; doi:10.3390/ijms19124012)
Supplement: Supplementary file 1 [file ijms-19-04012-s001.pdf]

Supplementary table 1. SAXS data collection and experimental parameters for the yeast proteins Sdo1, EFL1 and the complex EFL1•Sdo1.

**Data collection parameters**				
Beamline	B21, Diamond Light Source, Harwell (UK)			
Detector	Pilatus 2M			
Beam size	0.2 x 0.2 mm			
Energy	12.4 keV			
Sample to detector distance (mm)	4014			
q range ($Å^{-1}$)	0.0038–0.42			
Exposure time for frame (s)	3			
Number of frames	580			
Temperature (K)	293			
Mode	SEC online			
Beamline	P12, DESY/EMBL, Hamburg (Germany)			
Detector	Pilatus 2M			
Beam size	0.2 x 0.12 mm			
Energy	10.0 keV			
Sample to detector distance (mm)	3000			
q range ($Å^{-1}$)	0.0038–0.42			
Exposure time for frame (s)	1			
Number of frames	1850			
Temperature (K)	293			
Mode	SEC online			
**Structural parameters**				
	**Sdo1**	**Sdo1 2-3**	**EFL1**	**EFL1•Sdo1**
Concentration mg mL^{-1}	10	15.0	6.5	8
q interval for Fourier inversion ($Å^{-1}$)	0.015–0.21	0.015–0.18	0.01–0.18	0.012–0.15
R_g [from P(r)] (Å)	27 ± 0.5	20.6 ± 0.1	46 ± 0.6	51 ± 2.5
R_g [from Guinier approximation] (Å)	27.4 ± 0.5	20.45 ± 0.14	45.5 ± 1.0	47 ± 1.0
sRg limits [from Guinier approximation]	0.35–1.30	0.31–1.30	0.34–1.26	0.44–1.21
D_{max} (Å)	86	68	157	160
Porod volume estimate (nm^3)	42	25	258	333
DAMMIF excluded volume (nm^3)	52	31	350	378
Molecular mass (kDa)				
From Porod (x 0.53)	22	13	136	176
From excluded volume (x 0.5)	26	16	157	189
From sequence	29	18	127	156
Modelling				
Ambiguity	2.89 (highly ambiguous)	2.77 (highly ambiguous)	1.68 (may be ambiguous)	1.6 (may be ambiguous)
Resolution (Å)	22 ± 2	41 ± 3	34 ± 3	38 ± 3
**Software employed**				
Primary data reduction	DAWN pipeline (Diamond Light Source, UK)			
Data processing	ScÅtter v3.1q/ATSAS			
Ab initio modelling	DAMMIF/ GASBOR			
Validation and averaging	DAMAVER/DAMCLUST			
Model flexibility	MultiFOXS			
Computation of model intensities	CRYSOL			

$q = 4\pi sin(\theta/\lambda)$, where 2θ is the scattering angle and λ is the wavelength.

Supplementary table 2. Ensemble organization of the top 100 best scoring *state n* models represented by the SAXS data for the yeast proteins Sdo1 and EFL1.

	Conformation	R_g (Å)	Relative abundance (%)	Scoring function (χ)
		**Sdo1**		
1-state model	1	26.9	100	1.138
2-state model	1	28.5	58	1.138
	2	25.0	42	
3-state model	1	28.1	57	1.137
	2	24.2	34	
	3	30.4	9	
4-state model	1	28.1	33	1.137
	2	25.6	32	
	3	28.8	26	
	4	24.3	9	
		**EFL1**		
1-state model	1	41.7	100	2.59
2-state model	1	40.5	70	2.41
	2	45.1	30	
3-state model	1	40.2	57	2.40
	2	45.1	38	
	3	39.4	6	
4-state model	1	40.3	58	2.40
	2	45.1	23	
	3	45.6	13	
	4	39.4	6	

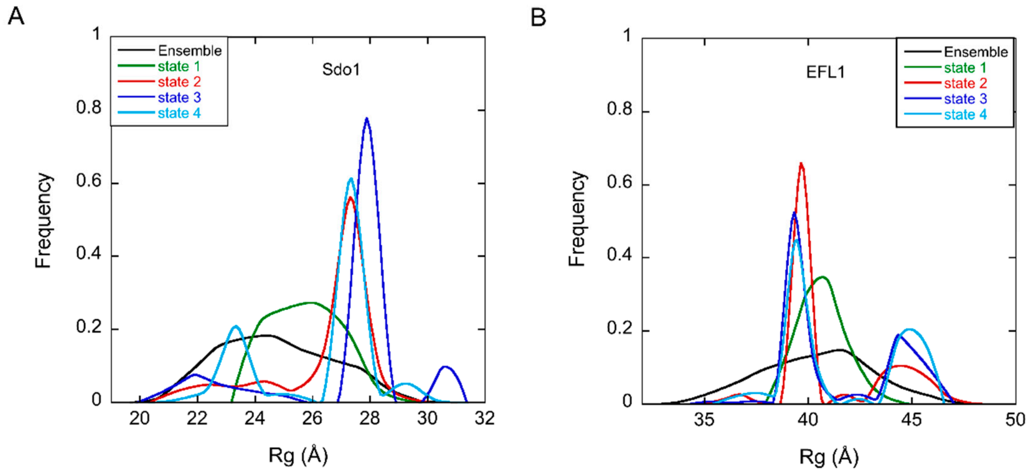

Supplementary figure 1. Distribution of the radius of gyration (R_g) in the initial pool of random structures of Sdo1 (**A**) and yeast EFL1 (**B**) overlapped to those of the final conformational sub-ensembles.

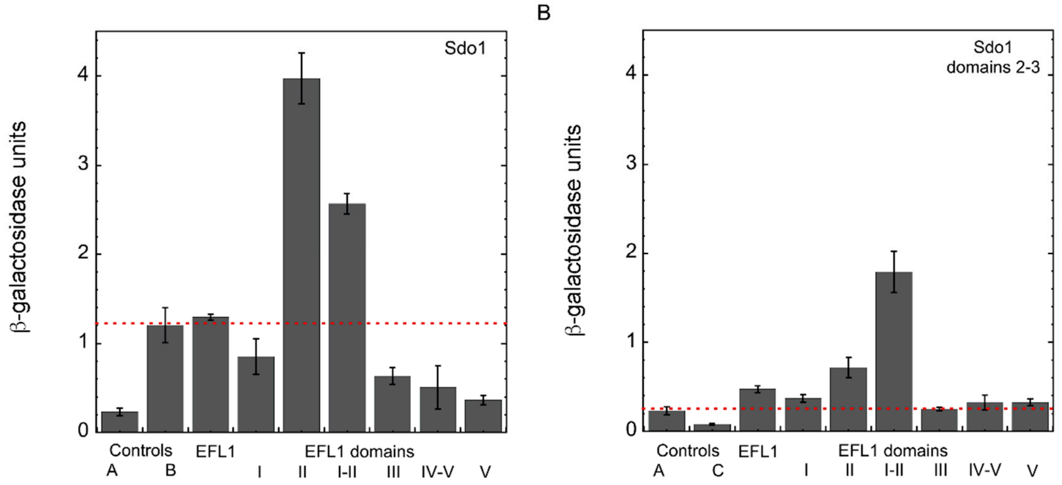

Supplementary figure 2. Binding of the wild type Sdo1 (**A**) and a construct of Sdo1 consisting of domains 2-3 (**B**) to the different domains of yeast EFL1 evaluated by yeast two hybrid. Control A - prey and bait empty vectors; Control B – empty pray vector and wild-type Sdo1 bait vector; Control C - empty pray vector and Sdo1 domains 2-3 bait vector. Columns represent the average of the three independent measurements while error bars represent the standard deviation.
